# Rheumatic manifestations in diabetic patients

**Published:** 2012-09-25

**Authors:** AL Serban, GF Udrea

**Affiliations:** * "C.I. Parhon" National Institute of Endocrinology, Bucharest; ** Department of Rheumatology and Internal Medicine, "Carol Davila" University of Medicine and Pharmacy, "I Cantacuzino"

**Keywords:** rheumatic condition, diabetes mellitus, musculoskeletal structure

## Abstract

Diabetes mellitus (DM), a worldwide high prevalence disease, is associated with a large variety of rheumatic manifestations. For most of these affections, pathophysiologic correlations are not well established. Some of them, such as diabetic cheiroarthropathy, neuropathic arthritis, diabetic amyotrophy, diabetic muscle infraction, are considered intrinsic complications of DM. For others, like diffuse idiopathic skeletal hyperostosis or reflex sympathetic dystrophy, DM is considered a predisposing condition. In most cases, these affections cause pain and disability, affecting the quality of life of diabetic patients, but once correctly diagnosed, they often respond to the treatment, that generally requires a multidisciplinary team. This article reviews some epidemiological, clinical, diagnostic and therapeutic aspects of these conditions.

## Introduction

Diabetes mellitus (DM) is a chronic disease characterized by an increased concentration of glucose in the blood, that occurs when the pancreas does not produce enough insulin or when the body cannot effectively use the insulin it produces. The prevalence of diabetes for all age groups worldwide was estimated to be of 2.8% in 2000 and 4.4% in 2030. The total number of people with diabetes is projected to rise from 171 million in 2000 to 366 million in 2030 [**1**].This increasing prevalence will inevitably result in an increasing prevalence of the diabetes and associated conditions including rheumatic manifestations. 

The wide spectrum of rheumatic affections related to DM can be classified according to the involved musculoskeletal structures. The rheumatologic manifestations of diabetes mellitus are the following: syndromes of limited joint mobility: diabetic hand syndrome (diabetic cheiroarthropathy), adhesive capsulitis (frozen shoulder, periarthritis), trigger finger (flexon tenosynovitis) dupuytren's contractures, osteoporosis); diffuse idiopathic skeletal hyperostosis (DISH); neuropathies: neuropathic arthritis (Charcot joints, diabetic osteoarthropathy, Carpal tunnel syndrome, diabetic amyotrophy, reflex sympathetic dystrophy, various other neuropathies; diabetic muscle infarction [**2**].

The conditions mentioned above can be divided into two main categories according the role of DM in the pathogeny of the condition: I. Primary role – the condition is considered an intrinsic complication of DM: Diabetic cheiroarthropathy, Neuropathic arthritis, Diabetic amyotrophy, Diabetic muscle infarction. II. Secondary role – DM is a predisposing condition: osteoporosis, reflex sympathetic dystrophy, etc.

**I. Syndromes of limited joint mobility** mainly involve upper limb musculoskeletal structures and seem to be associated with diabetes duration, poor metabolic control and presence of microvascular complications [**3**].

**Diabetic cheiroarthropathy**, or "stiff-hand syndrome" is characterized by painless limitation of mobility of the small joints of the hands. The prevalence ranges from 8% to 50% among patients with diabetes, compared with only 4% to 20% among individuals without DM [**4,5**]. Diabetic cheiroarthropathy is primarily a clinical diagnosis and the imaging findings are nonspecific [**6**]. There are two clinical sign which are essential for the diagnosis: prayer sign (the patient is unable to approximate the palmar surface of the fingers when raising the hands as if in prayer) and the tabletop sign (when the patient is asked to lay the palms flat on the tabletop he is unable to touch the palmar surface of the fingers to the table). The sonography findings of diabetic cheirotrapathy are the thickening of the flexor tendon sheaths and subcutaneous tissues [**6**], and IRM shows the thickening and enhancement of the flexor tendon sheaths [**7**]. The early recognition of this affection is important for two reasons: can be reversed by treatment and, in the same time it represents the marker of other diabetic microvascular complications. In a study by Rosenbloom et al. [**8**] the prevalence of proteinuria and retinopathy was of 11% in diabetic patients without diabetic cheiroarthropathy versus 50% in diabetic patients with diabetic cheiroarthropathy. As therapeutic measures, we include glycaemic control, non-steroidal antinflammatory drugs and physiotherapy. Surgery will reduce pressure on trapped nerves and improve sensation and discomfort, although some residual problems may remain.

**Dupuytren contracture (DC)** is characterized by the thickening and shorting of the palmar fascia, causing a contracture in flexion of the affected finger. In non-diabetic patients, the most affected fingers are the fourth and the fifth, but in individuals with DM, DC mainly affects third and fourth fingers and the hand involvement is frequently bilateral [**9**]. The prevalence of DC in diabetes ranges between 20% and 63%, higher than among subjects without diabetes, 13% [**4,10**]. DC is associated with diabetes duration, long-term poor metabolic control and presence of microvascular complications [**11**]. Diabetic cheiroarthropathy and Dupuytren contracture may coexist in the same patient [**4**]. Treatment of DC includes a good glycemic control, physiotherapy, topical steroid injection, and for the refractory cases surgery. Generalized hand stiffness has been observed after the surgical intervention [**12**].

**Trigger finger (stenosing flexor tenosynovitis)** is caused by the inflammation and subsequent narrowing of the A1 pulley, which causes finger blocking in flexion with the active extension failure. The middle and index fingers are the most commonly involved. The classic presentation of popping and locking of a trigger finger is usually sufficient for the diagnosis. There is no role for imaging in diagnosis, with X-rays considered unnecessary in patients without a history of inflammatory disease or trauma [**13**]. The prevalence of trigger finger ranges between 5% and 36% among patients with type 1 and 2 DM as compared with 2% in the general population [**14**]. The incidence of these disorders in diabetic subjects is associated with actual duration of the disease, not with glycemic control [**15**]. Trigger finger treatment consists in the modification of the activities to avoid the triggering of the digits, nonsteroidal anti-inflammatory drugs, splinting, corticosteroid injection into the tendon sheath and surgical release [**16**].

**Adhesive capsulitis (frozen shoulder)** is a condition characterized by an insidious and progressive loss of active and passive mobility of glenohumeral joint, presumably due to the capsular contraction [**17**]. The estimated prevalence of this condition is of 11-30% in diabetic patients, which is considerably greater than in non-diabetics [**4**]. Diabetes of long duration treated with insulin was associated with a larger percentage of shoulder calcification [**18**]. The diagnosis of adhesive capsulitis is often one of exclusion. Early in the disease process, adhesive capsulitis may clinically appear similar to other shoulder conditions such as major trauma, rotator cuff tear, rotator cuff contusion, labral tear, bone contusion, subacromial bursitis, cervical or peripheral neuropathy, previous surgical procedure. If a history of these other conditions is negative and if radiographs do not demonstrate osteoarthritis, then it could be adhesive capsulitis [**19**]. The treatment includes physical therapy, AINS, corticosteroid injection into the glenohumeral joint and subacromial bursa [**20**] and kinetotherapy.

**II. Osteoporosis** can occur in the diabetic patient as a direct consequence of the disease, but it can be also a treatment manifestation. The results of Nord Torndelag in Norway showed significant increase of hip fracture among women with type I DM compared with non-diabetic women. (relative risk=6.9, CI=2.2-21.6) [**21**]. Regarding type II diabetes, this association is not yet very well established. It was demonstrated that oral antidiabetic agents, the thiazolidinediones cause a bone mass decrease and a risk fracture increase [**22**].

**III. Diffuse idiopathic skeletal hyperostosis (DISH) (Forestier' Disease)** is associated with ligamentous ossification of the anterolateral aspect of the spinal column, sometimes leading to bony ankylosis. It was demonstrated that DISH is associated with diabetes mellitus particularly with non-insulin dependent diabetes. Several other metabolic disturbances and concomitant diseases have been suggested to be associated with DISH including obesity, increased waist circumference, hypertension, dyslipidemia, hyperuricaemia, metabolic syndrome [**23-26**]. Insulin has been proposed as a factor that promotes growth in DISH. In one study, patients with DISH and those with osteoarthritis had elevated levels of insulin and growth hormone, however, the level of IGF-1 was higher in patients with DISH than in those with osteoarthritis [**27**]. DISH is often an asymptomatic condition, but numerous clinical symptoms have been described including pain, stiffness, limited range of spinal motion and an increased susceptibility to unstable spinal fractures after trivial trauma. Cervical and lumbar segments of the spine are also frequently affected by DISH, and clinical manifestations include dysphagia and airway obstruction at cervical levels and radiculopathy. The diagnosis of DISH is based on radiologic features. Radiographic criteria for the diagnosis proposed by Resnick and Niwayama require the involvement of at least four contiguous thoracic vertebral segments, preservation of intervertebral disc spaces and the absence of apophyseal joint degeneration or sacroiliac inflammatory changes [**28**]. In 1985, Utsinger proposed a revised diagnostic criterion that incorporated the involvement of peripheral entheses. He suggested that symmetric peripheral enthesopathy and continuous ossification along with the anterolateral aspect of the 2 or more contiguous vertebral bodies support a probable diagnosis of DISH [**29**]. Treatment is generally symptomatic including analgesics, NSAIDs, local applications and physiotherapy. Control of associated metabolic disorders, may reduce the morbidities associated with these disorders, may retard future cardiovascular disease and possibly slow down the progression of soft tissue ossification. Therapeutic interventions should also aim at a reduction of insulin secretion and insulin resistance. 

**IV. Neuropathies**

**1. Neuropathic arthritis (Charcot joints, diabetic osteoarthropathy)** is a condition characterized in its early stages by acute inflammation that leads to bone and joint fractures, dislocation, instability and gross deformities [**30**]. In patients with diabetes, Charcot osteoarthropathy is associated with a longstanding duration of diabetes and peripheral neuropathy. The prevalence estimated among diabetic patients varies from 0.08 to 13% [**31**].

**Stage 0 (inflammation)**, is characterized by erythema, edema and heat but there are no structural changes visible on plain X-Ray [**32**].

**Stage 1 (development)** is characterized by bone resorption, bone fragmentation, and joint dislocation. The swelling, warmth, and redness persist, but there are also radiographic changes such as evidence of debris formation at the articular margins, osseous fragmentation, and joint disruption.

**Stage 2 (coalescence)** involves bony consolidation, osteosclerosis, and fusion after bony destruction. Absorption of small bone fragments, fusion of joints, and sclerosis of the bone are noticeable.

**Stage 3 - reconstruction of the damaged joints and bone** Healing and new bone occur. Decrease sclerosis and bony remodeling signify that the deformity (subluxation, incongruity and dislocation) is permanent [**33**].

The diagnosis is based on clinical features, laboratory tests and imaging studies. Clinical features include erythema, warmth, foot deformity, a medical history of long-standing diabetes. Radiographic aspects are important in diagnosing Charcot neuroarthropathy, although they are not present in patients with stage 0 disease. For this stage, magnetic resonance imaging, scintigraphy, white blood cells count are used, but MRI offers the highest diagnostic accuracy [**34**]. The aspects of MRI include ligamentous disruption, joint deformity, center of signal enhancement within joints and subchondral bone [**35**]. The goal of the treatment for acute or quiescent Charcot neuroarthropathy should be to maintain or achieve structural stability of the foot and ankle, to prevent skin ulceration, and to preserve the plantigrade shape of the foot so that prescription footwear can be used. Bisphosphonates can significantly reduce the levels of the bone turnover, temperature and pain, but the clinical benefit such as an earlier return to ambulation or radiographic improvement is weak at best. Surgery is reserved for severe ankle and midfoot deformities that are susceptible to skin ulceration and that make braces and orthotic devices difficult to use [**36**].

**2. Carpal tunnel syndrome (CTS)** is a painful disorder caused by the compression of the median nerve between the carpal ligament and other structures within the carpal tunnel. It has been reported in up to 20 percent of diabetic patients, but the incidence rises to 75 percent in those with limited joint mobility [**37**]. CTS may be more common in those with prediabetes [**38**]. The clinical features include irritative symptoms, such as nocturnal paresthesias, spontaneous pain, characterized by proximal irradiation, the ”shaking sign” (disappearance of the symptoms after vigorous flapping of the hands), the Tinnel sign, triggered by the percussion of the carpal tunnel, the patient reports pain resembling an electric sensation along the cours of the median nerve and, the Phalen test - the patient has to hold the hands against each other in full palmar flexion, paresthesias beginning between 30 to 120 s in this position. One essential parameter of the motor or sensory deficit is a pathological change in motor or sensory conduction velocity evaluated with the aid of electromyography. The treatment of CTS consists in conservative measures or surgical procedures. The conservative therapy includes splinting, steroids, activity modification, non-steroidal anti-inflammatory drugs, vitamin B6 and others. Of the conservative approaches, only splinting [**39**] and steroids are supported by high quality evidence [**40**]. Surgical release of the carpal tunnel is typically used in patients who fail to achieve an adequate relief with conservative managements and for those with moderate to severe symptoms [**41**]. However, in diabetic patient the recuperation after the surgical intervention is slower and less important [**42**].

**3. Diabetic amyotrophy** is a condition occurring in type I and II DM, in which patients develop severe aching or burning and lancinating pain in the hip and thigh, followed by a weakness and wasting of the thigh muscle and significant weight loss. It is associated with poor glycemic control [**43**]. The results of the electrodiagnostic studies, which are often met, are consistent with the presence of a neurogenic lesion that involves lumbosacral roots, plexus and peripheral nerves [**44**]. This condition is most likely caused by inflammatory, immune-mediated vascular radiculoplexopathy [**45,46**]. The diagnosis is based on a clinical presentation, the presence of diabetes and neural studies. Good functional recovery is expected within 2-3 years. Occasional relapses can occur. Medical therapy includes immunosuppressive agents, such as cyclophosphamide and methylprednisolone. Kifoyle et al. studied the therapy with pulsed methylprednisolone and noted a significant improvement in pain and weakness in the majority of the patients [**47**]. A Cochrane review concluded that no evidence from randomized trials supports any recommendation on the use of immunotherapy treatment in lumbosacral plexopathy [**48**]. The treatment also consists in a good glycemic control, physical therapy and occupational therapy.

**4. Reflex sympathetic dystrophy** is a component of complex regional pain syndrome (CRPS), a neuropathic pain disorder with significant autonomic features. CRPS is divided into CRPS-I (reflex sympathetic dystrophy) and CRPS II (causalgia) reflecting, respectively, the absence or the presence of documented nerve injury. CPRS I typically develops after a minor tissue trauma or bone fracture and is associated with a predisposing condition [**49**]. Diabetes mellitus and other endocrine (hyperthyroidism, hyperparathyroidism) and metabolic disease (IV hyperlipidemia) are predisposing conditions [**50**]. It has classic neuropathic pain characteristics (intense burn pain, hyperalgesia, allodynia), it is associated with local edema and changes suggestive of autonomic involvement (altered sweating, skin color and skin temperature in the affected region). The International Association for Study of Pain (IASP) [**51**] listed the diagnostic criteria for CRPS I. These criteria were improved by Bruehl [**52**] and Veldman [**53**] (**Table 1**). While IASP criteria are non-specified, possibly not as reproducible as Bruehl' s or Veldman' s criteria, they are cited more widely in the literature, including treatment trials [**54**]. The treatment includes medical therapy, invasive procedures and paramedical therapy. The medical therapy consists in analgesics, corticosteroids, oral muscle relaxants, bisphosphonates, and calcium-channel blockers. Invasive treatment consists in intravenous sympathetic blockade percutaneous sympathetic blockade, surgical sympathectomy, spinal cord stimulation, amputation. Paramedical interventions include physiotherapy, occupational therapy, and psychological treatment [**55**].

**Table 1:** Diagnostic criteria for CRPS I (Bruehl and Veldman)

IASP 1994 consensus criteria [**51**]	Bruehl's criteria [**52**]	Veldman’s criteria [**53**]
Criteria 2, 3 and 4 are necessary for a diagnosis of CRPS type 1. 1. Type 1 is a syndrome that develops after an initiating noxious event. 2. Spontaneous occurrence of pain in the absence of an external stimulus, allodynia (pain due to a mechanical or thermal stimulus that normally does not provoke pain), or hyperalgesia (exaggerated response to a stimulus that is normally painful) that is not limited to the territory of a single peripheral nerve, and is disproportionate to the inciting event. 3. There is or has been evidence of edema, skin blood flow abnormality, or abnormal sudomotor (sweating) activity in the region of the pain since the inciting event. 4. This diagnosis is excluded by the existence of conditions that would otherwise account for the degree of pain and dysfunction.	Continuing pain disproportionate to any inciting event. 1. Patient must report at least 1 symptom in each of the 4 following categories a) sensory: reports of hyperesthesia b) vasomotor: reports of temperature asymmetry or skin color changes or skin color asymmetry c) sudomotor/edema: reports of edema or sweating changes or sweating asymmetry d) motor/trophic: reports of decreased range of motion or motor dysfunction (weakness, tremor, dystonia) or trophic changes (hair, nail, skin) 2. Must display at least 1 sign in 2 or more of the following categories e) sensory: evidence of hyperalgesia (to pinprick) or allodynia (to light touch) f) vasomotor: evidence of temperature asymmetry or skin color changes or asymmetry g) sudomotor/edema: evidence of edema or sweating changes or sweating asymmetry h) motor/trophic: evidence of decreased range of motion or motor dysfunction (weakness, tremor, dystomia) or trophic changes (hair, nail, skin)	1. Presence of 4 out of 5 symptoms: a) Diffuse pain during exercise b) Temperature differences between affected and unaffected extremity c) Color differences between affected and unaffected extremity d) Volume differences between affected and unaffected extremity e) Limitations in active range of movement of the affected extremity 2. Occurrence or increase of symptoms during or after use 3. Symptoms in an area larger than the area of the primary injury

**V. Diabetic muscle infarction (DMI)** is an uncommon complication of diabetes that was first described by Angervall [56]. Since then, more than 100 cases have been reported. More than 50% of the reported cases had type I DM with a mean duration of 15 years [**57,58**]. Usually, DMI has an acute onset characterized by muscle pain and swelling. Thigh muscles are most frequently involved, however, calf muscle, simultaneous thigh and calf muscle and upper extremity muscle involvement have also been described. The diagnosis consists in the presence of long standing diabetes, clinical aspects and imaging tests. MRI is the diagnostic test of choice. MRI gives a typical picture on T2-weighted images, with marked muscle oedema extending into the perifascicular and subcutaneous tissues [**59**]. Muscle biopsy should be reserved for patients with atypical clinical presentation, those in whom the diagnosis is uncertain and those who do not improve with antiplatelet or anti-inflammatory drug therapy [**60**]. Differential diagnosis is made with myositis, venous thrombosis, tumor and diabetic amyotrophy, adverse effect of simvastatin, ruptured Baker's Cyst. The recommended management of this condition is symptomatic, with pain relief and short-term immobilization as necessary. This condition tends to resolve over a period of weeks to a month in most cases. Optimizing diabetic control is of paramount importance [**61**]. Medical therapy with antiplatelet or anti-inflammatory drugs is recommended for DMI, but no randomized controlled trial has been performed because of the infrequent occurrence of this condition [**58**].This condition recurs in about half of those affected [**57**].
